# Transcranial Direct Current Stimulation Over the Left Dorsolateral Prefrontal Cortex Reduced Attention Bias Toward Negative Facial Expression: A Pilot Study in Healthy Subjects

**DOI:** 10.3389/fnins.2022.894798

**Published:** 2022-06-20

**Authors:** Shuang Liu, Siyu Zhai, Dongyue Guo, Sitong Chen, Yuchen He, Yufeng Ke, Dong Ming

**Affiliations:** ^1^Academy of Medical Engineering and Translational Medicine, Tianjin University, Tianjin, China; ^2^School of Precision Instruments and Optoelectronics Engineering, Tianjin University, Tianjin, China

**Keywords:** tDCS, DLPFC, attention bias, ERP, emotion regulation

## Abstract

Research in the cognitive neuroscience field has shown that individuals with a stronger attention bias for negative information had higher depression risk, which may be the underlying pathogenesis of depression. This dysfunction of affect-biased attention also represents a decline in emotion regulation ability. Clinical studies have suggested that transcranial direct current stimulation (tDCS) treatment can improve the symptoms of depression, yet the neural mechanism behind this improvement is still veiled. This study aims to investigate the effects of tDCS on affect-biased attention. A sample of healthy participants received 20 min active (*n* = 22) or sham tDCS (*n* = 19) over the left dorsolateral prefrontal cortex (DLPFC) for 7 consecutive days. Electroencephalographic (EEG) signals were recorded while performing the rest task and emotional oddball task. The oddball task required response to pictures of the target (positive or negative) emotional facial stimuli and neglecting distracter (negative or positive) or standard (neutral) stimuli. Welch power spectrum estimation algorithm was applied to calculate frontal alpha asymmetry (FAA) in the rest task, and the overlapping averaging method was used to extract event-related potentials (ERP) components in the oddball task. Compared to sham tDCS, active tDCS caused an obvious increment in FAA in connection with emotion regulation (*p* < 0.05). Also, participants in the active tDCS group show greater P3 amplitudes following positive targets (*p* < 0.05) and greater N2 amplitudes following negative distracters (*p* < 0.05), reflecting emotion-related attention biases. These results offer valuable insights into the relationship between affect-biased attention and the effects of tDCS, which may be of assistance in exploring the neuropathological mechanism of depression and anxiety and new treatment strategies for tDCS.

## Introduction

Initiated in automatic or controlled ways, selective attention refers to the cognitive processes that serve to filter relevant external and internal information for further processing (Stevens and Bavelier, 2012). In the context of emotional processing, selective attention is a crucial mechanism that influences our emotional experience and functioning by determining how the organism perceives and interprets emotional information in the environment (Gross et al., 2011; Sanchez et al., 2015). Attention bias is a sort of selective attention that occurs when individuals have high sensitivity to certain specific information (Bar-Haim et al., 2007). Studies have suggested that individual differences in attention bias underlie difficulties in emotion regulation processes, especially for depressed people (Joormann and D’Avanzato, 2010). Healthy individuals under negative emotion states transfer their attention more frequently to positive stimuli (Sanchez et al., 2014) and take attention away more rapidly from negative stimuli (Ellenbogen et al., 2002). However, individuals suffering from anxiety and depression show negative biases in selective attention (Armstrong and Olatunji, 2012). For instance, signal detection studies (Wiens et al., 2008) have found that individuals with anxiety disorders have lower detection thresholds for threatening stimuli, and research on spatial attention suggests that these decreased thresholds lead to increased orienting toward threats Mogg et al., 2002), which indicates that patients with anxiety disorders show attentional bias toward threats. This negative bias may increase state anxiety by causing increased awareness of threats (Armstrong and Olatunji, 2012). Besides, a small number of studies have observed reduced attention toward positive stimuli in depressed individuals relative to controls (Peckham et al., 2013). More seriously, people susceptible to depression have been characterized as attending to and remembering negative information (Siegle et al., 2002). This attentional style could lead to distorted beliefs and assumptions about the world (Armstrong and Olatunji, 2012).

The negative bias could be seen as reduced responsiveness or drive to engage with positive stimuli from surroundings, namely a positive attenuation effect. Alternatively, negative bias has been interpretable in terms of overwhelming attention to negative information, which can be thought of as a negative potentiation effect (Rottenberg et al., 2005). The attention bias toward negative stimuli would be related to a lack of inhibition of negative material, resulting from faulty inhibitory processes of interference control (Waters et al., 2006). Attentional bias mechanisms may be particularly active and impactful when processing socioemotional information, such as others’ emotional facial expressions (Gotlib et al., 2004a). Facial expressions are among the most commonly perceived visual stimuli and transmit and evoke emotion simultaneously. For instance, an attention bias toward sad or threatening faces more or less increases one’s negative emotions and leads to terrible representations in social communication (Frewen and Dozois, 2005). One of the hallmarks of depression is impaired social function, and previous research has documented that this manifestation stems from different attentional biases toward different facial expressions (Persad and Polivy, 1993).

Many behavioral studies have shown that there are some differences in the attention to different emotional faces between depressed and healthy groups. These findings can correspond to and verify the above hypotheses. Depressed groups gazed and engaged attention into negative faces for a longer time (Fritzsche et al., 2010), and were more likely to attend to negative facial expressions in a series of facial stimuli with different valence (Karparova et al., 2010). Even more, healthy subjects were more inclined to pay attention to negative faces after negative emotions’ induction (Kujawa et al., 2011). Some articles also reported that depressed groups attended to positive information insufficiently, manifesting that they pay less attention to happy faces than sad faces in the same conditions (Suslow et al., 2004). In general, depressed groups showed abnormally larger attention bias toward negative facial expressions compared with healthy controls.

Cognitive neuroscience has provided a new perspective on revealing brain mechanisms behind psychological phenomena theoretically and technically. There is an increase in the research on underlying neural correlates of attention biases toward facial expressions *via* neuroscience methods such as the event-related potentials (ERP) technique, which can uniquely complement traditional behavioral measures. Emotional facial expressions can evoke different ERP components well, some of that are related to attention processing (Schindler and Bublatzky, 2020). The P1, N1, and P2 belong to exogenous ERP components, which are easily affected by stimulus characteristics due to the early automatic attention mechanism, reflecting the bottom-up processing of attention. N2 and P3 are endogenous ERP components, occurring in later stages of processing and are closely related to cognition, reflecting the top-down processing of attention (Hopfinger and West, 2006; Brosch et al., 2011). Previous studies have reported that the abnormality of attention may be caused by the inefficient engagement of top-down control (Friedman-Hill et al., 2010; Delchau et al., 2019). Therefore, examining P3 and N2 could help discover the underlying neural processes behind attention biases to understand attention and inhibition behaviors better.

A new viewpoint noted that affect-biased attention is considered to be a form of emotion regulation (Bram et al., 2018). Growing evidence indicates that depression is always accompanied by dysregulation of emotion (Berking et al., 2014; Vanderlind et al., 2020). With the development of measuring methods in brain activities, frontal alpha asymmetry (FAA) has been increasingly seen as a reliable index of the capability of emotion regulation. FAA examines the activity of the left and right frontal alpha waves (typically at F3 and F4 electrodes). The intensity of alpha wave activity is inversely proportional to the intensity of activity in the corresponding cortical region. A strong alpha wave represents a weak activity in the brain and vice versa (Kline et al., 2007; Zhong et al., 2011). The left and right prefrontal cortex hemispheres show different attention biases to different emotional stimuli. The left frontal cortical activity is associated with positive effects, while the right frontal area might be susceptible to negative emotions (Davidson, 1998; Harmon-Jones et al., 2010). It is believed that individuals with high emotional regulation ability generally have greater left frontal activity (Dagmar Kr et al., 2010). The increase in left frontal activity tends to enhance the positive emotional experience (Tomarken et al., 1992). Anxiety and depressive symptoms caused by mood regulation disorders are associated with decreased left frontal lobe activity (Smit et al., 2007).

Neuroimaging studies demonstrate an interactive network of corticolimbic pathways playing a central role in the top-down regulation of emotions (Johnstone et al., 2007; Wager et al., 2008). Specifically, a functional balance between ventral [ventral anterior cingulate cortex (ACC), limbic structures] with dorsal compartments in the brain [dorsal ACC, dorsolateral prefrontal cortex (DLPFC)] is necessary for maintaining homeostatic emotional control (Ochsner and Gross, 2005). These brain structures are also involved in the attentional processing of emotional information (Fragopanagos et al., 2005), with the DLPFC as an important region for the implementation of top-down attentional control (MacDonald Angus et al., 2000). Therefore, emotional attentional biases associated with the dysregulation of emotional states can be understood as the result of failures in top-down attentional control implemented by the DLPFC (De Raedt et al., 2015). Therefore, the variation of activities in DLPFC might be associated with the formation of depression. Clinical studies have suggested that lesions of the left DLPFC are often associated with depression, while damages in the right DLPFC lead to elevated mood (Schmitz et al., 2006). Hypoactivity of the left DLPFC is thought to play a key role in the pathophysiology of depression, sometimes accompanied by increased right DLPFC functioning (Schutter and Honk, 2005; Hecht, 2010).

In recent years, transcranial direct current stimulation (tDCS) has shown promise as a neuromodulatory tool to study neuropsychological functioning (Shin et al., 2015). Constant low-intensity direct current (typically 0.5–2 mA) is applied to modulate spontaneous cortical activity. The application of current produces polarity-specific subthreshold changes in the excitability of underlying targeted cortical areas. Research has demonstrated that anodal stimulation increases cortical excitability, whereas cathodal stimulation decreases cortical activity (Nitsche and Paulus, 2001; Nitsche et al., 2003). To date, tDCS has been reported to significantly modulate a range of cognitive and affective abilities in healthy participants and patients with depression (Boggio et al., 2009; Wolkenstein and Plewnia, 2013; Salehinejad et al., 2016; Yadollahpour et al., 2017). However, few results have yielded the mechanism of tDCS. Combined with the importance of emotional attentional bias on patients, we hypothesized that tDCS could modulate the affective abilities of subjects by improving the negative cognitive bias.

In this study, we aimed to disentangle the effect of prefrontal tDCS on attention bias toward emotional information and emotion regulation abilities in healthy subjects. Considering the advantages of high temporal resolution and low cost, we used EEG to record the changes of cortical activity after tDCS to explore the neural correlation of attention towards emotional information. This study contained a rest task and a modified emotional oddball task consisting of six blocks, which could response attention bias. EEGs were recorded during rest and oddball task phases, respectively. Hereby, we selected healthy subjects rather than depressed patients to exclude the influence of clinical treatments such as antidepressant medications. Therefore, this study could be regarded as a preliminary attempt to investigate how appropriate tDCS protocol works in the treatment of depression. Since the main purpose is to explore the effects of tDCS in the treatment of depression, given the above-mentioned importance of the left DLPFC in depression, we performed only left DLPFC anodal stimulation in this study.

## Materials and Methods

### Participants

A total of forty-one healthy participants (25 females; mean age = 23.41) were included in this study. The healthy participants were college students or graduate students of Tianjin University, meeting the following criteria: (1) right-handed; (2) aged 18–25; (3) Chinese native; (4) no history of neurological disorders or brain injuries; and (5) no metal object and implantable devices in brain. The healthy participants were randomly assigned into two groups: 22 in the active tDCS group and 19 in the sham tDCS group.

The study protocol was approved by the Tianjin University, and all investigative procedures were conducted according to the principles expressed in the Declaration of Helsinki. Written informed consent was obtained after the nature of the procedures was explained and before any study procedures.

The healthy participants need to complete the questionnaires to measure the anxiety level and emotion regulation ability twice before and after tDCS. The anxiety level was assessed with the State-Trait Anxiety Inventory (STAI) (Spielberger, 1970) and emotion regulation ability was assessed with the Difficulties in Emotion Regulation Scale (DERS) (Gratz and Roemer, 2008).

### Procedures

**FIGURE 1:**
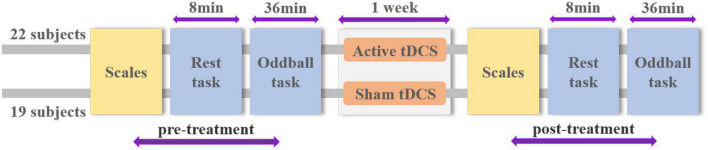
Schematic overview of experimental design and procedure.

The study was conducted on 7 consecutive days and involved three stages: pre-treatment test, stimulation, and post-treatment test. Day 1 started with the pre-treatment test phase consisting of rest and oddball tasks. Then all participants underwent 20 min active or sham tDCS over the left DLPFC for 7 consecutive days. On day 7, after the last time of stimulation, the participants completed the post-treatment test phase with the same contents as the pre-treatment. Figure 1 shows the overall flow of the experiment. EEG data were acquired in both pre- and post-treatment test phases. In addition, participants filled out the STAI and DERS questionnaires before EEG recording. The whole experiment was executed using Matlab 2013b with Psychtoolbox 3.0.11 (Brainard, 1997).

#### Rest Task

**FIGURE 2:**
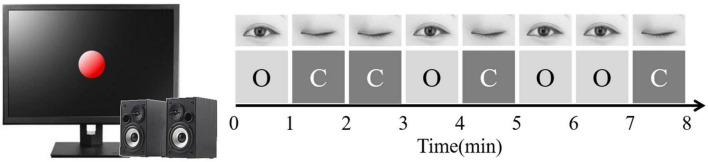
Rest task procedure. “O” means “open,” “C” means “closed.”

The participants were seated in front of a computer monitor and instructed to be relaxed in a sound-attenuated laboratory. The recorded voice prompts guided subjects to open (O) and close (C) their eyes during the 8-min rest task. A red circle appeared in the center of the screen as a visual reference point when the rest task started. In the meantime, their EEG signals were collected. The rest task contained eight 1-min intervals in the order of “OCCOCOOC” (see Figure 2). We averaged across eyes open and eyes closed conditions because both were highly correlated (*r* = 0.83, *p* < 0.001). Spearman–Brown corrected reliability was 0.91 for the eyes open and eyes closed conditions and the average of the two conditions could produce a more reliable estimate of frontal asymmetry than either single condition (Hagemann, 2004; Tomarken et al., 2010). Split half reliability between the first and second 4 min of data assessment of 0.98 indicates excellent reliability and suggests stability of measurement at least for our 8 min of data recording.

#### Experimental Stimuli

Human faces with different emotions were planned to use as stimulus materials. But research has shown that there is a racial bias in human face recognition, i.e., people can better recognize the facial expressions of their countries of ethnic groups (McAndrew, 1986). To avoid the interference of stimulus materials, emotional faces were selected from the Chinese Facial Affective Picture System (CFAPS) (Bai et al., 2005), including 162 positive faces, 162 negative faces, and 126 neutral faces. Male and female faces have an equal number in the chosen pictures.

#### Oddball Task

Participants were seated in front of the monitor, and all relevant instructions were shown on the computer screen initially. The three-stimulus visual oddball paradigm was applied in the session. The session comprised six blocks, and each oddball task block had 180 trials. Each trial began with a fixed white cross appearing in the center of the screen for 250 ms, and then a facial stimulus was presented for 750 ms with the interstimulus interval of 1,000 ms. In each block, the order of the 180 trial presentations was pseudo-random with overall proportions of 70% frequent standards, 15% rare targets, and 15% rare distracters. The targets were separated by at least one non-target stimuli.

Before the experiment began, the participants were allowed to be familiar with a short practice block. For the formal experiment, they were required to focus on the middle of the screen and respond by hitting the space bar on the keyboard as quickly as possible to present target stimuli during oddball tasks.

**FIGURE 3:**
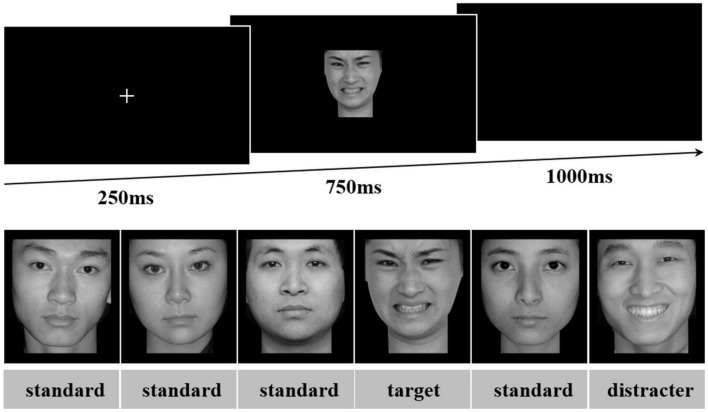
Oddball task. A single trial of the oddball task is shown, with the fixation, stimulus, and blank screen sequence depicted. A sequence of stimuli that could be presented is shown with trial-type labels added in the figure for easier identification. This sequence would be within a block in which negative faces served as targets. Source for the photos: the Chinese Face Affective Picture System.

In the session, each participant completed three blocks in which positive faces were targets and three blocks in which negative faces were targets. The opposite emotional valence served as distracters (e.g., negative distracters in the block with positive targets), while neutral faces were the frequently presented standard stimuli. Each block had 27 positive, 27 negative, and 126 neutral facial expression stimuli. Each target and distracter stimulus was presented only once during the whole oddball task. Within each task block, the gender of faces was balanced. Each task block lasted 6 min, and participants were given short breaks between blocks. The block order was counterbalanced across subjects. The schematic experimental procedure of the oddball paradigm is illustrated in Figure 3.

### Transcranial Direct Current Stimulation

Transcranial direct current stimulation was delivered by a battery-driven constant current stimulator (DC-Stimulator PLUS, NeuroConn GmbH, Ilmenau, Germany) using a pair of rubber electrodes in a 5 × 5 cm saline-soaked synthetic sponge. The anode was placed over the left DLPFC (electrode position F3) and the cathode was placed over the right DLPFC (electrode position F4) according to the international 10–20 EEG system. According to healthy subjects, for active tDCS, a constant current of 1.5 mA for 20 min was applied with a gradual ramp up/down of the current over the first and last 30 s, respectively. For sham stimulation, the current ramped up to 1.5 mA within 15s at the beginning of the stimulation and then ramped down within 15s later. The protocols that applied from 1 to 2 mA of current (current density: 0.28–0.57 A/cm^2^) for 20 min between 5 and 15 sessions have been demonstrated to be safe (Jasper et al., 1958; Loo et al., 2009, 2012; Palm et al., 2012).

### Electrophysiological Data Recording and Preprocessing

During the experiment, 64 channels EEG signals were recorded continuously using a Neuroscan 4.5 amplifier system. The electrodes were placed on the scalp according to the extension of the international 10–20 electrode positioning system. The electrode impedances were kept below 5 KΩ. All signals were amplified with a 0.05–100 Hz band-pass and sampled at the rate of 1,000 Hz, using a right mastoid reference electrode.

Offline analysis was performed using EEGLAB Toolbox (Delorme and Makeig, 2004), which are open-source Matlab packages for EEG analysis. The EEG signals were re-referenced to the average of the bilateral mastoid electrodes. The signals were resampled to 200 Hz and low-pass filtered with 45 Hz.

Eye blinks and movement artifacts were eliminated with independent components analysis (ICA). EEG waveforms were time-locked to each stimulus onset and were segmented from 200 ms before the stimulus onset to 1,000 ms after stimulus onset. All epochs were baseline-corrected with respect to the mean voltage over the 200 ms preceding the stimulus onset. Trials containing activity exceeding ±80 μV at any site were excluded from averaging.

### Data Analysis

In the study, we calculate the FAA values based on EEG signals of the rest task. The ERP waveforms were acquired when the stimuli appeared, and collected behavioral data were performed for statistical analysis in the oddball task.

#### Frontal Alpha Asymmetry

Welch spectrum energy spectrum estimation of alpha was applied to calculate the value of FAA. This study used the pwelch function in Matlab to realize the power spectrum estimation of the resting EEG data. The 1–45 Hz frequency band of each lead of the 60-lead EEG data is divided into five frequency bands for power spectral density estimation. The definitions of the bands are as follows: delta (1–4 Hz), theta (4–8 Hz), alpha (8–12 Hz), beta (12–30 Hz), and gamma (30–45 Hz). In this experiment, the EEG data were segmented every 5 s as a sample and the power spectrum of each band sample of each lead was calculated. The power value of the power spectrum of each lead in a specific frequency band was the sum of each frequency point in the band. The method to calculate the FAA value is to record the alpha wave intensity of the left frontal lobe and the right frontal lobe EEG at rest, and then calculate the natural logarithm of the alpha wave intensity of the right frontal lobe electrode point and the left frontal lobe. The FAA indexes were obtained by subtracting the two natural logarithms (right minus left): ln (R) – ln (L).

#### Behavioral Data

For behavioral data, trials were excluded if the reaction time was shorter than 200 ms or longer than 1,500 ms. The data whose average value was greater than or less than the average of all participants in each group plus/minus three times the variance would not be adopted during statistics. The paired-sample *t*-test was used to analyze the behavioral data of each group before and after treatment, and the independent-sample *t*-test was used to perform the statistical analysis between the two groups.

The changes in the reaction time, accuracy, and errors of the commission have naturally become the focus of behavioral results. The reaction time was calculated by correct trials of the oddball task, representing the speed of attentional processing under targeted emotional stimuli. The accuracy shows whether the subject responded correctly to targets, indicating the accurate identification of targeted emotional stimuli. These two types of behavioral data reflect the attentional bias to different emotional stimuli, which indicate faster attention occupancy and more accurate attention recognition (Most et al., 2005). Errors of commission are referred to the proportion of incorrect button responses to task-irrelevant stimuli including distracters and neutral standards. The commission errors of distracters include two types: taking negative as positive when targets are defined positive, and taking positive as negative when targets are defined negative. The commission errors of neutral standards include taking neutral as positive and neutral as negative.

#### Event-Related Potentials

For ERP data, the average amplitudes were overlaid for correct trials in the three positive blocks and negative blocks. This study focused on the P3 and N2 components elicited by positive, negative, and neutral facial stimuli in two groups. For each participant, the target P3 value consisted of the mean amplitude from standard trials subtracted from the mean amplitude of target trials. The distracter N2 value was derived by subtracting standard trials from distracter trials. To isolate the primary ERP components associated with attention to emotional facial expressions, a traditional windowed analysis was conducted on individual average files. The P3 amplitude was calculated at the Pz electrode site between 400 and 600 ms. To examine the N2 component, the 250–450 ms temporal window and FCz location were selected.

## Results

### Questionnaire Data

Validity test was conducted on the collected questionnaires and invalid questionnaires were eliminated. Since incomplete filling questionnaires, 20 were effectively received in the active tDCS group and 15 in the sham tDCS group. The STAI could calculate the state anxiety and trait anxiety scores, while the DERS showed the emotion regulation score. The STAI analysis proved that all subjects’ state and trait anxiety before and after the experiment belonged to the normal range. The DERS analysis found no significant difference in the emotion regulation ability of the subjects before and after the experiment in both the groups (*p* > 0.05). The state anxiety and trait scores and emotion regulation ability scale scores are shown in Table 1.

**TABLE 1:** Mean score of two groups by STAI and DERS.

Group	Testing time	State anxiety (M ± SD)	Trait anxiety (M ± SD)	Emotion regulation (M ± SD)
**Active group**				
	Pre-treatment	31.35 ± 7.99	38.50 ± 8.03	75.50 ± 14.25
	Post-treatment	35.10 ± 8.48	37.30 ± 7.98	72.83 ± 16.04
**Sham group**				
	Pre-treatment	34.00 ± 6.51	41.60 ± 9.47	75.36 ± 18.84
	Post-treatment	31.40 ± 6.70	37.20 ± 7.51	75.21 ± 20.48

### Behavioral Data

#### Reaction Time

For the active group, the average reaction time of the positive targets in the pre- and post-treatment tests was 601 ± 44 and 607 ± 57 ms, while the reaction time of the negative targets was 604 ± 43 and 611 ± 45 ms. For the sham group, the average reaction time of positive targets in the pre- and post-treatment tests was 618 ± 76 and 618 ± 54 ms, while the reaction time of negative targets was 631 ± 78 and 619 ± 54 ms. But there were no significant differences between the pre- and post-treatment tests in both the active and sham groups (*p* > 0.05).

#### Accuracy

For the active group, the accuracy of positive targets in the pre- and post-treatment tests was 84.8 ± 11.8% and 86.6 ± 9.9%, while the accuracy of negative targets was 89.3 ± 9.4% and 87.8 ± 11.4%. For the sham group, the accuracy of positive targets in the pre- and post-treatment tests was 85.5 ± 7.8% and 88.6 ± 7.4%, while the accuracy of negative targets was 89.2 ± 7.9% and 90.7 ± 5.8%. But there were no significant differences between the pre- and post-treatment tests in both the active and sham groups (*p* > 0.05).

#### Commission Error

The commission errors of distracters between the pre- and post-treatment tests in the active and sham groups had no significant differences (*p* > 0.05). For the commission errors of neutral standards, significant differences were found between the two phases test and two groups (see Tables 2, 3).

**TABLE 2:** The commission errors rates of neutral standards between pre- and post-treatment test in two groups.

Group	Type	Pre-treatment (M ± SD)	Post-treatment (M ± SD)	*P*-value
**Active group**				
	Neu as Pos	0.212 ± 0.359	0.178 ± 0.329	0.329
	Neu as Neg	0.256 ± 0.377	0.156 ± 0.276	0.011*
**Sham group**				
	Neu as Pos	0.106 ± 0.314	0.042 ± 0.037	0.385
	Neu as Neg	0.053 ± 0.079	0.047 ± 0.079	0.599

“Neu” means “Neutral,” “Pos” means “Positive,” “Neg” means “Negative.” *P < 0.05.

**TABLE 3:** The commission errors rates of neutral standards differences before and after treatment between two groups.

Type	Active group (M ± SD)	Sham group (M ± SD)	*P*-value
Neu as Pos	–0.035 ± 0.159	0.008 ± 0.032	0.248
Neu as Neg	–0.101 ± 0.161	–0.006 ± 0.048	0.020*

“Neu” means “Neutral,” “Pos” means “Positive,” “Neg” means “Negative.” *P < 0.05.

### Frontal Alpha Asymmetry Data

In the previous studies, FAA mostly applied the scores of the dorsal position such as F4-F3, F6-F5 as the main indicator, and some studies also used the FP2-FP1 score of the frontal position as the asymmetry index. Here, we calculated asymmetry coefficients at the four paired-electrodes of F4-F3, F6-F5, F8-F7, and FP2-FP1.

Table 4 shows the asymmetry coefficients of the pre- and post-treatment tests at the four paired electrodes in the active/sham groups. Paired *t*-test was used to examine the differences in FAA between the pre- and post-treatment in the active/sham group. The asymmetry coefficients of F4-F3 and F6-F5 positions increased significantly after tDCS in the active group (*p* = 0.025, *p* = 0.041) and no significant differences were found at the F8-F7 and FP2-FP1 positions (*p* > 0.05). There were no significant differences in the asymmetry coefficients at the four paired electrodes in the sham group.

**TABLE 4:** The asymmetry coefficients in different paired-electrodes before and after treatment in two groups.

Group	Paired-electrodes	Pre-treatment value (M ± SD)	Post-treatment value (M ± SD)	*P*-value
**Active group**				
	F4-F3	–0.290 ± 0.650	0.164 ± 0.402	0.025*
	F6-F5	–0.304 ± 0.712	0.199 ± 0.473	0.041*
	F8-7	0.081 ± 0.745	0.342 ± 0.556	0.189
	FP2-P1	–0.107 ± 0.862	0.064 ± 0.620	0.462
**Sham group**				
	F4-3	–0.063 ± 0.453	–0.164 ± 0.620	0.497
	F6-5	0.041 ± 0.626	–0.028 ± 0.589	0.949
	F8-7	–0.221 ± 0.958	–0.390 ± 0.915	0.576
	FP2-P1	–0.067 ± 0.786	–0.112 ± 0.585	0.809

*P < 0.05.

To further verify the differences between the two groups, an independent *t*-test was performed to analyze the values in the pre-treatment test and difference values (post-treatment value minus pre-treatment value) of the active and sham groups. For asymmetry coefficients in the pre-treatment test, no significant difference was found in the four paired electrodes between the two groups (*p* > 0.05). It indicated that subjects in different groups had no baseline difference, thus the changes between the two groups after treatment were comparable. The asymmetry coefficient difference of the active group was greater than that of the sham group at the F4-F3 and F6-F5 positions, but significance was found only in the F4-F3 position (see Table 5).

**TABLE 5:** The asymmetry coefficients differences before and after treatment between two groups.

Paired-electrodes	Active group (M ± SD)	Sham group (M ± SD)	*P*-value
F4-F3	0.454 ± 0.810	–0.101 ± 0.600	0.026*
F6-F5	0.504 ± 1.004	0.013 ± 0.826	0.120

### Event-Related Potentials Data

**FIGURE 4:**
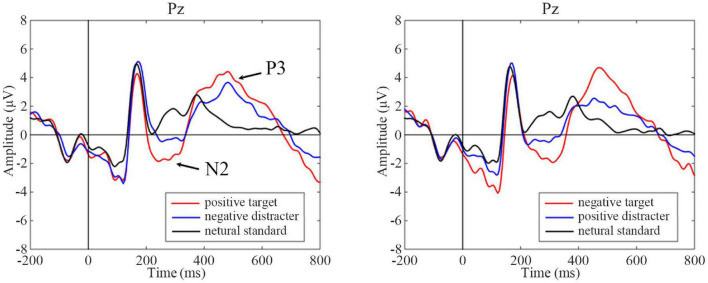
Event-related potentials (ERP) waves on different task conditions at pre-treatment test in the sham group as an illustration. Black vertical lines indicate the initial time, and P3 and N2 components are marked with arrows. Positive target task shows on the left and negative target task on the right. For ERP plots, the *y*-axis displays amplitude (μV) and the x-axis displays time (ms).

The difference in the ERP amplitude in the oddball paradigm reflects the different attention levels to standard and deviant stimulus. As this experiment included three types of stimulus (targets, distracters, and standards), the ERP waveforms of different stimulus types were examined at first. Referring to other studies using the oddball paradigm, the waveform changes at the midline leads are mainly preferred. Pz was selected here to draw the ERP waveforms when the positive and negative emotional faces are targets, and they were shown in Figure 4, respectively. For ERP components, this article focused on the changes in the P3 and N2 components before and after tDCS. The original ERP waveform after tDCS in the active group was taken as an example here. It can be seen from Figure 4 that the amplitudes of P3 and N2 were subject to targets > distracters > standards, which conformed to the waveform distribution of the three-stimulus type oddball paradigm.

To study the effect of tDCS on attention biases for different emotional faces, the ERP amplitude changes of positive and negative targets (or distracters) before and after tDCS in the active/sham tDCS group were compared, respectively. To avoid the impact of other properties on the ERP of targets (or distracters), the ERP difference wave that was equal to the targets (or distracters) minus the standard reported value was used to present the results, for the same rules might exist in some ERP components between targets (or distracters) and standards.

#### P3 Event-Related Potentials Data

**FIGURE 5:**
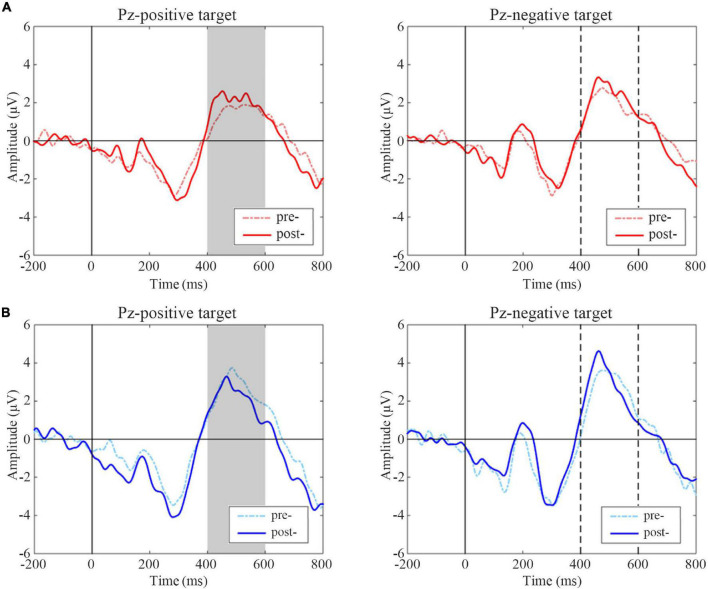
Event-related potentials (ERP) difference waves of pre- and post-treatment tests for positive and negative targets in two groups. **(A)** Active group, **(B)** Sham group. The gray-filled area indicates the period with a significant difference. The gray dotted lines indicate the time points at 400 and 600 ms.

A statistical analysis of ERP difference wave amplitudes was performed using a paired *t*-test, and the results are shown in Figure 5. The post-treatment test showed greater amplitude in the P3 window than the pre-treatment test inactive group following positive targets (*t* = –2.294, *p* = 0.032) but not negative targets (*t* = –1.107, *p* = 0.281). A significant decrease in positive targets was observed in the P3 time window in the post-treatment test compared with the pre-treatment test in the sham group (*t* = 2.184, *p* = 0.042); while there were no significant changes in the P3 amplitudes of negative targets in the sham group (*t* = –0.771, *p* = 0.451).

#### N2 Event-Related Potentials Data

**FIGURE 6:**
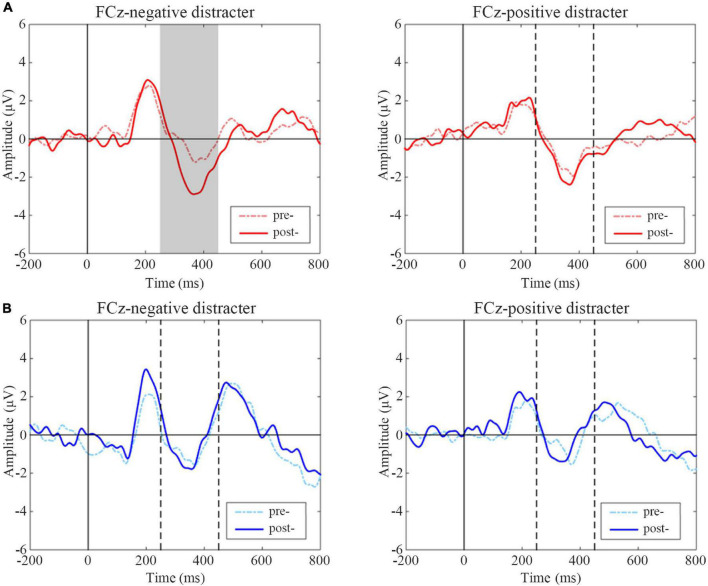
Event-related potentials (ERP) difference waves of pre- and post-treatment tests for negative and positive distracters in two groups. **(A)** Active group, **(B)** Sham group. The gray-filled area indicates the period with a significant difference and the gray dotted lines indicate the time points at 250 and 450 ms.

N2 difference waveform shifts of two groups are shown in Figure 6. Subjects in the active group had significantly increased amplitudes of negative distracters on the condition of positive targets after treatment (*t* = 2.508, *p* = 0.020), while there was no significant change of positive distracters on the condition of negative targets (*t* = –0.578, *p* = 0.570). In sham group, there was no significant difference between pre- and post-treatment on two conditions (*t* = –0.381, *p* = 0.707; *t* = 0.518, *p* = 0.610).

## Discussion

Recently, many researchers generally agreed that affect-biased attention is a form of emotion regulation. Also, affect-biased attention is tuned through experience over development (Todd et al., 2012), and individuals with different personality traits tend to show different attention biases (Paelecke et al., 2012). Several studies have suggested that depressive patients show a stronger attention bias for negative information, which may be related to the severity of depressive symptoms (Gotlib et al., 2004b; Sanchez et al., 2013). TDCS could improve depression and anxiety behaviors shown in clinical studies using enhancing cognitive control (Regni et al., 2010; Peña-Gómez et al., 2011). Few studies have explored the influence of tDCS on attentional bias. Thus, this study investigated attentional processing before and after tDCS within a healthy sample. TDCS reduced the commission error of taking neutral as negative, but there were no significant differences in the reaction time and accuracy. We found enhanced frontal alpha asymmetry coefficients after active tDCS treatment. The ERP results showed greater P3 amplitudes following positive targets and greater N2 amplitudes following negative distracters in the active tDCS group.

For behavioral data, only commission error of taking neutral as negative was significantly decreased after tDCS treatment and results including reaction time as well as accuracy showed no significant differences. It must be mentioned that all of the participants were healthy college students. It was not difficult for them to complete the simple oddball task. Therefore, we speculated there might exist the ceiling effect, resulting in the barely noticeable difference before and after tDCS treatment. And that is why we designed this study and analyzed the electrophysiological signals of the subjects. With high-time resolution, ERP could react to the real variations of subjects during the oddball task.

The P3 is thought to indicate processes involved in salient stimulus and is known to be modulated by attention (Becker and Shapiro, 1980; Heinze et al., 1990). The P3 amplitude has further been used to measure attentional allocation of cognitive processing resources in multiple tasks (Polich and Kok, 1995; Sawaki and Katayama, 2008). Affective stimuli can induce P3 signals well, and negative stimuli evokes greater P3 amplitudes than positive stimuli in healthy subjects (Morita et al., 2001). Compared to healthy groups, depressed individuals showed an enhanced P3 response to negative stimuli and a smaller P3 for positive stimuli (Ilardi et al., 2007). This can be seen as increased attention to the negative and a failure to attend sufficiently to the positive. However, many behavioral and psychophysiological findings showed that attention deficiency is widespread in anxiety, depression, and even attention deficit hyperactivity disorder (ADHD). Reduced P3 amplitude for task-relevant stimuli were observed in depressed groups as compared to the controls (Singh et al., 2000). In this study, we found increased P3 amplitudes following positive targets in the active group after receiving tDCS. This result suggests that tDCS may improve the allocation of attention to task-relevant positive stimuli in a healthy sample. P3 signals evoked by positive targets decreased significantly in the sham group. It is probably because the subjects’ attention to positive stimuli declined after being familiar with the paradigm.

Our results also provide somewhat evidence to suggest that tDCS may affect response inhibition to negative emotional stimuli in a healthy sample. In previous electrophysiological studies, the fronto-central N2 component has traditionally been interpreted as an index of response inhibition (Falkenstein et al., 1999; Falkenstein, 2006). Response inhibition research commonly applied Go/Nogo paradigms, which require the execution of a motor response on a Go stimulus and its inhibition on a Nogo stimulus (Kaiser et al., 2006). Decreased N2 amplitude has been implicated in a failure of inhibiting or task-irrelevant disinhibition. Accumulating evidence demonstrated that depression is associated with difficulty in inhibiting negative information. Some studies found abnormally decreased N2 amplitude following task-irrelevant or distracting negative affective pictures or facial stimuli in the depressed group (Joormann and Gotlib, 2008; Krompinger and Simons, 2009). From a general cognitive functioning perspective, this might be negative bias in attentional processing resulting in inhibitory deficits in the depressed group (Botvinick et al., 2001). We observed increased N2 following negative distracters significantly in the active group after treatment. Nevertheless, no significant amplitude changes were observed following positive distracters as well as in the sham group. Such difference before and after treatment could be explained in that tDCS may improve response inhibition to negative information in healthy subjects.

Besides, previous research on frontal lobe EEG lateralization found that frontal lobe EEG lateralization is associated with depression, anxiety, schizophrenia, aggressiveness, and other emotional and behavioral disorders (Aftanas and Pavlov, 2005; Mathersul et al., 2008; Reznik and Allen, 2017). A recent research’s result showed that frontal EEG lateralization can be used as a neurological indicator of depression (Gollan et al., 2014). Individuals with anxiety and depression have weakened left frontal lobe activity, while individuals with high emotional regulation ability have stronger left frontal lobe activity (Goodman et al., 2013). Thus, the implementation of emotional regulation and the use of emotional regulation strategies have enhanced left frontal lobe activity (Choi et al., 2016). In this study, we found that the FAA values of F4-F3 and F6-F5 positions increased significantly after tDCS in the active group, indicating the elevated emotion regulation ability.

Additionally, the DLPFC is of great importance in the top-down regulation of affective processing and highly correlated to emotion regulation (Baeken et al., 2010). Some studies have shown that tDCS has regulatory effects on DLPFC activity (Boggio et al., 2009) maybe by mediating cerebral blood flow and metabolism (Shiozawa et al., 2015). Anodal tDCS targeting the left DLPFC has reported significant antidepressant effects and improvement in emotional cognitive control, while tDCS over the right DLPFC leads to the generation of attentional impairments (Sanchez et al., 2016, 2018). A recent tDCS study showed a lateralized role of left and right DLPFC activity in enhancing/worsening the top-down regulation of emotional attention processing (Allaert et al., 2019). Our findings from two tasks were consistent with the above conclusions, illustrating the probable link between affect-bias attention and emotion regulation. But through Pearson correlation analysis and the mediating effect analysis, we did not find out the explicit link between ERP waveforms and FAA values statistically.

Some limitations should be considered in this study. First, our results could only reflect the effects of tDCS on electrophysiological data, while the results of behavioral data could not support the electrophysiological results. That means that the conclusions of this study are still inconclusive. TDCS could affect the subjects’ ERP and FAA, but the relationship between these results and emotional attention bias is still uncertain. In the above discussion, we only provide a possible explanation direction that these electrophysiological results may reflect attentional bias. Second, this study employed three types of emotional facial stimuli, i.e., positive, negative, and neutral, to analyze the affect-biased attention in emotional processing. However, we have made a basic classification of emotional stimuli only in terms of valence roughly. The effect of tDCS is unknown when the emotional stimulus is classified as multiple categories and dimensions in more fine-grained details. Third, our results should be considered in the light of our sample. We recruited a restricted sample from a young college student population with an education level above average. Thus, it remains to be elucidated whether results from this study generalize to other groups. Additionally, the results of the correlation analysis between FAA and ERP did not show the exact link between emotional processing and the functional connection of the prefrontal areas. Nonetheless, considering the underlying mechanism, future studies should think about how to explain the regulation of emotional attention by the prefrontal cortex better physiologically.

In sum, our findings offered some electrophysiological insight into how tDCS works in the treatment of depression. TDCS treatment may raise the level of attention allocation to the positive target stimulus, reduce the negative cognitive bias, and enhance emotion regulation ability. To some extent, results of ERP in oddball task and FAA in rest task may reflect the improvement of affect-bias attention and emotional regulation ability after tDCS. The above conclusions were only evidenced by physiological data significantly, excluding the inaccuracy and deception of self-assessment. Despite the limitations, this study adds to our understanding of changes that occurred in the brain region and may support the rationale for new therapies based on neuromodulation techniques. Future research is needed to replicate, extend, and refine these findings in depressed or dysphoric individuals to explore the feasibility of tDCS application toward emotional disorders.

## Data Availability Statement

The raw data supporting the conclusions of this article will be made available by the authors, without undue reservation.

## Ethics Statement

The studies involving human participants were reviewed and approved by the Ethics Committee of Tianjin Hospital, Tianjin University. The patients/participants provided their written informed consent to participate in this study. Written informed consent was obtained from the individual(s) for the publication of any potentially identifiable images or data included in this article.

## Author Contributions

SL and DM conceived of the presented idea. SL designed the experiment. SZ performed the experiments. YH performed the computations. DG and SC verified the analytical methods. YK and DM helped supervise the project. All authors discussed the results and contributed to the final manuscript.

## Conflict of Interest

The authors declare that the research was conducted in the absence of any commercial or financial relationships that could be construed as a potential conflict of interest.

## Publisher’s Note

All claims expressed in this article are solely those of the authors and do not necessarily represent those of their affiliated organizations, or those of the publisher, the editors and the reviewers. Any product that may be evaluated in this article, or claim that may be made by its manufacturer, is not guaranteed or endorsed by the publisher.
